# A panoramic view of hospitalized young children in the metropolitan area of the valley of Mexico during COVID-19

**DOI:** 10.1016/j.ijregi.2023.10.004

**Published:** 2023-10-12

**Authors:** Gerardo R. Padilla-Rivas, Michelle G. Santoyo-Suarez, Diego Francisco Benitez-Chao, Kame Galan-Huerta, Hector Franco Villareal, Elsa N. Garza-Treviño, Jose Francisco Islas

**Affiliations:** 1 Universidad Autónoma de Nuevo León, Facultad de Medicina, Departamento de Bioquímica y Medicina Molecular, Dr. Eduardo Aguirre Pequeño, Monterrey, México; 2 Althian Clinical Research, Capitán Aguilar Sur 669, Monterrey, México

**Keywords:** Mexico hospitalizations, Mexico COVID-19, Children COVID-19, COVID-19 death, COVID-19 comorbidities

## Abstract

**Objectives:**

This work provides an overview of young children's (aged 0-9) infectious diseases epidemiology, by exploring the link between various comorbid conditions, COVID-19, and death rate.

**Methods:**

Public data on hospitalized young children was obtained from national databases of the Mexican health care system from 2020-2022. Data included age, year of entry, gender, the time between admission to death (hospitalization time), date of death, comorbidities, and admissions to the intensive care unit. Children were separated into age groups and frequencies were calculated. Binary regression models were developed to determine the correlation of comorbidities and COVID-19 to death as calculated by odds ratios (OR).

**Results:**

From 2020-2022, there were 11,815 hospitalizations among young children, of which 15.98% were due to COVID-19, 2.55% of hospitalizations resulted in fatalities from which 32.45% of deaths were COVID-19 related. The highest case-calculated fatality ratio of COVID-19 infected young children was estimated at 7.04% by early 2020, but dropped to 2.11% by the end of the second semester of 2022. The most frequent comorbidities associated with their hospitalization and death for the general population were intubation (OR: 17.967), pneumonia (OR: 2.263), diabetes (OR: 7.301), cardiovascular diseases (OR: 1.528) and COVID-19 (OR: 261). For the COVID-19-positive group, the most impactful comorbidities were intubation (OR: 20.232), pneumonia (OR: 3.057), and diabetes (OR: 12.824).

**Conclusion:**

Children's hospitalizations and deaths were common during the pandemic; wherein major comorbidities played an important role. Therefore, effective comorbidity management and vaccination programs are essential to reduce hospitalizations and deaths among young children.

## Highlights

• A total of 1888 hospitalized children were reported as COVID-19 positive, with a mortality rate of 5.19%.
• In 2020, mortality reached its highest peak at 10.37% in young children.
• The study shows that 70% of the total children's deaths related to newborn infants with COVID-19.
• Newborn infants with COVID-19 represented 51.8% of the total inquiries.
• Mortality was amplified when intubated, had pneumonia, and/or diabetes in children.

## Introduction

Since the initial phases of the COVID-19 pandemic, Mexico, alongside other Latin-American countries such as Costa Rica, Peru, Brazil, Colombia, and the Caribbeans had some of the highest COVID-19 case fatalities in the world. As a region, Latin America had an estimated 1.7 million reported deaths related to COVID-19 by the end of September 2022, which represented just over a quarter of total cases worldwide; despite the region only having close to 8% of the world's population [1]. As an example, data for Costa Rica showed that between March 2020 and the end of 2021, COVID-19-related deaths represented 15% of total demises in the country [2]. Meanwhile, during the similar time period in Peru, official reports showed 188,708 deaths potentially related to COVID-19 [3]. In the particular case of México, COVID-19 case fatalities reached close to 10% by mid-2020 and maintained up to 9% in adults [2] with slightly lower percentages in the rest of these countries, making Latin America a highly vulnerable region to death by COVID-19 infections [4]. Noticeably, according to the World Health Organization (WHO) these numbers were believed to be underestimated, particularly as the region had struggled with the availability of diagnostic tests, problems accessing health services, hospital saturation, misclassifications, deaths attributed to other comorbidities, lack of adequate reporting, and more recently access to adequate vaccination programs [2,5].

Over time, numerous studies have arisen with a much better outlook on the general situation, principally as vaccines and less lethal COVID-19 variants and strains, such as Omicron have emerged [6]. Importantly, COVID-19 studies have given us insights into the virus's behavior (variants and mutations), as well as other important information such as vaccination effectiveness [4], yet most of these studies have been focusing on the adult populations [7], relegating the effects the virus could have on children. Interestingly, with the outbreak of the SARS-CoV-2 virus, leading to the COVID-19 disease, reports have often stated that children who may contract this infection tend to be asymptomatic, and with potentially good prognosis [8,9], even if no other comorbidities are present. Not surprisingly, original thoughts of children and COVID-19 infections seemed to point toward the direction that children were less likely to get infected, as well as to potentially spread the disease [10]. Even current reports have suggested that while COVID-19-related deaths in children have acquired ground in the U.S. although, infections continue low. Death rates for children in the U.S. were reported at around 1 per 100,000 for individuals ages 0-19, with the highest mortality rate, amongst children younger than 1 year (4.3 per 100,000), ranking COVID-19 as the eighth cause of death, which still only represents 2% of total deaths for individuals ages 0 to 19 [11].

The focus of this study is to analyze infections and mortality associated with the COVID-19 pandemic in Mexico, taking data from the metropolitan area of the valley of Mexico, the largest regional metropolitan area in North America, which represents approximately 20% of the country's population [12]. Our aim is to describe the complete panorama for hospitalized young children ages 0-9 years, as they represent some of the most vulnerable populations in terms of vulnerability to infections. To describe the panorama as it pertains to young children, information from national health databases was obtained. The analysis of the data as it relates to infection and, most importantly to mortality, while describing other conditions, can help draw better parameters to attend to young children in the management of future pandemics.

## Methods

Public data were obtained through the Mexico City Ministry of Health's portal "Datos abiertos" (https://datos.cdmx.gob.mx/). The data encompasses nearly 6 million registries on the Mexican health care system from 2020-2022. Particularly, the registry included 11,815 entries for hospitalized young children (between the ages of 0-9), from which 1888 children tested positive for COVID-19. The national registry included information on age, year of entry, gender, date of death (when applicable), and comorbidities such as cardiovascular diseases (CVD's), diabetes, hypertension, pneumonia, intubation, and others, as well as if admitted patients entered the intensive care unit (ICU). All National registry data includes a patient registry number, which does not link any entry to any patient information, as directed under federal data protection law “Ley de la protección de datos personales en posesión de sujetos obligados” (https://www.diputados.gob.mx/LeyesBiblio/pdf/LGPDPPSO.pdf), to help protect patient anonymity.

### Frequencies and relation to COVID-19 and Death

Frequency data were calculated for all variables in the registry; age was set in intervals of 0-2 years, 3-5 years, and 6-9 years. This included individual variable data for the generalized population, as well as for the COVID-19-specific segment. In both cases, data was further accounted for using death as a restrictive parameter and separated by year. In addition, we further determined the time between admission to death (hospitalization time), in critical time intervals 0-3 days, 4-7 days, 7-14 days, and above 15 days in relation to age groups.

### Binary logistic regression analysis

A binary logistic regression model was developed for both the general population and for the COVID-19 population to determine the influence of each comorbidity. Considering only significant comorbidities (*P*-values < 0,05), a second regression was developed. All statistical analyses, including binary logistic regression, were performed using IBM SPSS Statistics for (version 23.0) (IBM Corp., Armonk, NY, USA).

### Treemapping and parallel sets

Using statistically significant variables (*P*-values < 0.05), we calculated the percentage for the individual comorbidities and for the comorbidity's relation. Data is presented using hierarchical data Treemaps, and all non-significant variables and no comorbidities were grouped into a single variable. Additional visualization of entries was done using Parallel developed in RStudio v. 1.3.1093 with R v. 4.0.3, using the following packages: reshape2 v. 1.4.4, tidyverse v. 1.3.0, and ggforce v. 0.3.3. These packages can be downloaded from CRAN (https://cran.rproject.org/).

## Results

### General population and COVID-19 positive population statistics

The Mexico City Ministry of Health has been diligently recording entries from the public health care system, including certain entries from the private health sector. Since the official beginning of the COVID-19 pandemic in 2020 and throughout the end of 2022, there have been over 6 million individual entries. Particularly, 11,815 entries have accounted for hospitalized young children, with 1888 cases belonging to COVID-19-positive young children or 15.98% of the hospitalizations. Fortunately, out of the total hospitalization cases, there have only been 302 recorded deaths (2.52%), yet 32.45% of these deaths were deemed COVID-19 positive. Overall COVID-19-positive deaths within the COVID-19 population or case fatality ratio represented 5.19% (Table 1 and Table 2).

**Table 1:** General profile of hospitalized young children in the Mexican Health Care System.

Individual characteristics		General population		COVID-19-positive population	
		n = 11815	*P*-value	n = 1888	*P*-value
		n (%)		n (%)	
Gender					
	Female	5123 (43.36)	0.001	814 (43.11)	**0.819**
	Male	6692 (56.64)		1074 (56.89)	
by Age					
	0 - 2 years	6484 (54.88)	0.001	978 (51.80)	0.013
	3 - 5 years	2547 (21.56)		438 (23.20)	
	6 - 9 years	2784 (23.56)		472 (25.00)	
Intubated					
	No	11180 (94.63)	0.001	1774 (93.96)	**0.163**
	Yes	635 (5.37)		114 (6.04)	
Diabetes					
	No	11744 (99.40)	0.001	1870 (99.05)	0.049
	Yes	71 (0.60)		18 (0.95)	
Pneumonia					
	No	10390 (87.94)	0.001	1531 (81.09)	0.001
	Yes	1425 (12.06)		357 (18.91)	
Chronic obstructive pulmonary disease					
	No	11782 (99.72)	0.001	1875 (99.31)	0.001
	Yes	33 (0.28)		13 (0.69)	
Asthma					
	No	11593 (98.12)	0.001	1864 (98.73)	0.037
	Yes	222 (1.88)		24 (1.27)	
Immunosuppression					
	No	10806 (91.46)	0.001	1738 (92.06)	**0.323**
	Yes	1009 (8.54)		150 (7.94)	
Hypertension					
	No	11716 (99.16)	0.001	1863 (98.68)	0.015
	Yes	99 (0.84)		25 (1.32)	
Cardiovascular diseases					
	No	11254 (95.25)	0.001	1820 (96.40)	0.011
	Yes	561 (4.75)		68 (3.60)	
Obesity					
	No	11706 (99.08)	0.001	1859 (98.46)	0.004
	Yes	109 (0.92)		29 (1.54)	
Renal failure					
	No	11676 (98.82)	0.001	1871 (99.10)	**0.247**
	Yes	139 (1.18)		17 (0.90)	
COVID-19					
	Negative	9927 (84.02)	0.001	-	-
	Positive	1888 (15.98)		1888 (100)	
Intensive care unit					
	No	10819 (91.57)	0.001	1685 (89.20)	0.001
	Yes	996 (8.43)		203 (10.75)	
Death					
	No	11513	0.001	1790	0.001
	2020	4455 (38.70)		492 (27.49)	
	2021	3329 (28.92)		398 (22.23)	
	2022	3729 (32.39)		900 (50.27)	
	Yes	302	0.001	98	0.275
	2020	172 (56.95)		51 (52.04)	
	2021	85 (28.15)		28 (28.57)	
	2022	45 (14.90)		19 (19.39)

**Table 2:** Profile (death population) of hospitalized young children in the Mexican Health Care System.

Individual characteristics		General population		COVID-19-positive population	
		n = 302	*P*-value	n = 98	*P*-value
		n (%)		n (%)	
Gender					
	Female	141 (46.70)	**0.24**	47 (48.00)	**0.346**
	Male	161 (53.30)		51 (52.00)	
by Age					
	0 - 2 years	225 (74.50)	0.001	69 (70.40)	**0.315**
	3 - 5 years	32 (10.60)		10 (10.20)	
	6 - 9 years	45 (14.90)		19 (19.40)	
Intubated					
	No	144 (47.68)	0.001	48 (48.98)	**0.823**
	Yes	158 (52.32)		50 (51.02)	
Diabetes					
	No	289 (95.70)	0.001	89 (90.82)	0.006
	Yes	13 (4.30)		9 (9.18)	
Pneumonia					
	No	164 (54.30)	0.001	44 (44.90)	0.027
	Yes	138 (45.70)		54 (55.10)	
Chronic obstructive pulmonary disease					
	No	302 (100)	-	98 (100)	-
	Yes	-		-	
Asthma					
	No	301 (99.67)	0.048	97 (98.98)	**0.325**
	Yes	1 (1.33)		1 (1.02)	
Immunosuppression					
	No	272 (90.07)	0.406	90 (91.86)	**0.543**
	Yes	30 (9.93)		8 (8.16)	
Hypertension					
	No	292 (96.69)	0.001	91 (92.86)	**0.015**
	Yes	10 (3.31)		7 (7.14)	
Cardiovascular diseases					
	No	269 (89.07)	0.001	89 (90.82)	**0.56**
	Yes	33 (10.93)		9 (9.18)	
Obesity					
	No	294 (97.35)	0.007	96 (97.96)	**1**
	Yes	8 (2.65)		2 (2.04)	
Renal Failure					
	No	296 (98.01)	0.172	94 (95.92)	**0.089**
	Yes	6 (1.99)		4 (4.08)	
COVID-19					
	Negative	204 (67.55)	0.001	-	-
	Positive	98 (32.45)		98 (100)	
Intensive care unit					
	No	175 (57.95)	0.001	56 (57.14)	**0.8886**
	Yes	127 (42.05)		42 (42.86)	
Death					
	Yes	302	0.001	98	0.275
	2020	172 (56.95)		51 (52.04)	
	2021	85 (28.15)		28 (28.57)	
	2022	45 (14.90)		19 (19.39)	

The data shows a consistent gender distribution amongst young children in both the general and the COVID-19 populations (corresponding to 43.36% and 43.11%, in females). Newborn infants (0-2 years) were the group most affected in both general and COVID-19 patients (54.88% and 51.8% of total patients), this was followed by the oldest group 6-9 years which had the second highest number of hospitalizations and COVID-19 patients (23.56% and 25% of total patients). Interestingly, the newborn infants group represented over 70% of total deaths in both general and COVID-19 patients. It is also important to mention that other important comorbidities were recorded for young children. Out of these records pneumonia was the comorbidity with the highest percentage in both groups (12.06% and 18.91%), followed by immunosuppression (8.54% and 7.94%) and intubation (5.37% and 6.04%). Meanwhile, 8.43% of total patients were directly admitted to the ICU, which in the COVID-19-positive group represented 10.75% (Table 1).

One striking fact was seen in hospitalizations, which initially were reduced by a quarter from 2020-2021 going from 4455-3329 cases, which again rose by 12% from 3329 to 3729 when compared to the end of 2022. This growth phenomenon was even more remarkable in the COVID-19 cases, where in 2020, there were 492 reported cases, which dropped to 398 cases in 2021 but upsurged to 900 by 2022 (Table 1 and separated by age groups in Table 3).

**Table 3:** Profile of hospitalized young children. A relation of death by age range per year.

Death (Year)	Age range	Total population		Death population	
		General population	COVID-19-positive population	General population	COVID-19-positive population
No					
2020		4455 (*P* <0.001)	492 (*P* <0.009)		
	0 - 2 years	2521 (56.59)	295 (59.96)	-	-
	3 - 5 years	937 (21.03)	107 (21.75)	-	-
	6 - 9 years	997 (22.38)	90 (18.29)	-	-
2021		3329 (*P* <0.001)	398 **(*P* <0.238)**		
	0 - 2 years	1805 (54.22)	193 (48.49)	-	-
	3 - 5 years	686 (20.61)	81 (20.35)	-	-
	6 - 9 years	838 (25.17)	124 (31.16)	-	-
2022		3729 **(*P* <0.866)**	900 **(*P* <0.237)**		
	0 - 2 years	1933 (51.84)	421 (46.78)	-	-
	3 - 5 years	892 (23.92)	240 (26. 67)	-	-
	6 - 9 years	904 (24.24)	239 (26.56)	-	-
Yes					
2020				172 (*P* <0.001)	51 (*P* <0.001)
	0 - 2 years	-	-	132 (76.74)	39 (76.47)
	3 - 5 years	-	-	13 (7.56)	2 (3.92)
	6 - 9 years	-	-	27 (15.70)	19 (19.61)
2021				85 (*P* <0.001)	28 (*P* <0.001)
	0 - 2 years	-	-	66 (77.65)	18 (64.29)
	3 - 5 years	-	-	8 (9.41)	3 (10.71)
	6 - 9 years	-	-	11 (12.94)	7 (25.00)
2022				45 **(*P* <0.374)**	19 (*P* <0.008)
	0 - 2 years	-	-	27 (60.00)	12 (63.16)
	3 - 5 years	-	-	11 (24.44)	5 (26.32)
	6 - 9 years	-	-	7 (15.56)	2 (10.53)

### Death statistics and comorbidity significance

A part of our interest resides in surveying conditions related to death in young children. We began by observing that death was a variable that over time was reducing in both the general and the COVID-19-positive populations, as more than half of demises occurred in 2020 (56.95% and 52.04%), and nearly being reduced by half each consecutive year. With relation to comorbidities and death the highest percentages were seen in patients who were intubated (52.32% and 51.02%), as well as those with pneumonia (45.70% and 55.10%), followed by those with CVD's (10.93% and 9.18%). Moreover, just above 40% of these patients were directly admitted to the ICU (42.05% and 42.86%) (Table 2). When assessing deaths related to hospitalization time, just under 60% of passings occurred within a week (59.93% in the general population, and 58.16% in the COVID-19 population), with clearly a majority occurring within the first 3 days. The most affected population were newborn infants as they, out of all the groups, had the highest incidence of mortality at all different time intervals, averaging 74.50% in the general population and 70.40% in the COVID-19 population. This was followed by the 6-9 years group, which had an incidence of 14.9% and 19.39% (Table 4).

**Table 4:** Relation of admission - death (days) to children's age.

Population type	Admission -death (days)	0 - 2 years	3 - 5 years	6 - 9 years	Total	%
		n (%)	n (%)	n (%)		
General population						
	0 - 3 days	83 (74.11)	13 (11.61)	16 (14.29)	112	37.09%
	4 - 7 days	50 (72.46)	6 (8.70)	13 (18.84)	69	22.85%
	7 - 14 days	41 (69.49)	9 (15.25)	9 (15.25)	59	19.54%
	> 15 days	51 (82.26)	4 (6.45)	7 (11.29)	62	20.53%
	Total	225 (74.50)	32 (10.60)	45 (14.90)	302	100.00%
COVID-19 positive population						
	0 - 3 days	19 (67.90)	3 (10.70)	6 (21.40)	28	28.57%
	4 - 7 days	19 (65.50)	3 (10.30)	7 (24.10)	29	29.59%
	7 - 14 days	12 (63.20)	3 (15.80)	4 (21.10)	19	19.39%
	> 15 days	19 (86.40)	1 (4.50)	2 (9.10)	22	22.45%
	Total	69 (70.40)	10 (10.20)	19 (19.38)	98	100.00%

In order to further assess the complete panorama of both populations, binary logistic regression models were developed taking into consideration all comorbidities. Complete model results showed for the general population that intubation, pneumonia, diabetes, CVD's, and COVID-19 were all statistically significant. Meanwhile, in the COVID-19 population, CVD's did not seem to play a significant role (Supplemental Table 1). Taking these variables into consideration only, a second set of binary logistic regression models was run to find the adjusted odds ratio (OR) values, as for the general population intubation had the highest influence with OR: 17.967 followed by diabetes with an OR: 7.301. A similar pattern was also observed in the COVID-19-positive population, with intubation presenting an OR: 20.232 and diabetes OR: 12.824 (Table 5). A visual representation of the behaviors considering the significant comorbidities of both the general population and the COVID-19 population was grafted as parallel sets plots (Supplemental Figure 1). Furthermore, a hierarchical treemap was also developed showing the percentages of the significant comorbidities represented. All other comorbidities, including no comorbidity, were summed into a single variable (Supplemental Figure 2).

**Table 5:** Binary logistic regression analysis on hospitalized children.

Population type	Comorbidities	B	SE	Wald	DF	*P*-values	Odds ratio	95% confidence interval for odds ratio	
								Inferior	superior
General population^a^	Intubated	2,889	0.142	4,13,688	1	<0.001	17.967	13.602	23.734
	Pneumonia	0.817	0.142	33,124	1	<0.001	2.263	1.714	2.989
	Diabetes	1,988	0.386	26,521	1	<0.001	7.301	3.426	15.559
	Cardiovascular diseases	0.459	0.219	4,395	1	0.036	1.582	1.030	2.429
	COVID-19	0.959	0.14	46,871	1	<0.001	2..61	1.983	3.434
COVID-19 positive population^b^	Intubated	3,007	0.256	137.54	1	<0.001	20.232	12.240	33.443
	Pneumonia	1,117	0.247	20,442	1	<0.001	3.057	18.830	4.961
	Diabetes	2,551	0.588	18,847	1	<0.001	12.824	4.053	40.574

a Model summary: −2 log-likelihood = 2142.799. Cox and Snell R square = 0.055. and Nagelkerke R square = 0.260

b Model summary: −2 log-likelihood = 2132.283. Cox and Snell R square = 0.056. and Nagelkerke R square = 0.264

## Discussion

The COVID-19 pandemic has posed a significant threat to global health, including the well-being of children. For Mexico, our study spans from 2020-2022; a five COVID-19 wave period in which there was an increase in the number of hospitalizations due to the development of new variants and strains, yet an effective reduction in severity metrics, such as death which had its highest level during the winter of 2020 (second wave). We should mention that for Mexico the epidemic waves corresponded to the following periods: the first wave from March 29-October 03, 2020; the second wave corresponded to March 04, 20020 until May 29, 2021 (which had the highest number of individualized cases as well as the highest number of death patients); the third wave was from May 30 until December 18, 2021; the fourth wave was during the period of December 19, 2021-April 30, 2022; and finally, the fifth wave which covered the period of May 01-August 27, 2022 [13]. As mentioned, these periods represent the onset of COVID-19 at different times in Mexico. Understandably for other countries epidemiological waves occurred at different times, yet unsurprisingly other epidemiological studies found similarities, particularly during the winter of 2020 [1,2,11,14]. In recent studies focused on health care workers, researchers determined that the period of September to December 2020 (Italian third wave) had the highest COVID-19-hospitalization incidence, as well as death [14,15]. To counteract these effects, in many parts of Europe massive vaccination campaigns were set forth in action by the end of December 2020, ultimately resulting in a vast reduction both in hospitalization and death. At the Bari Policlinico University-Hospital researchers found that the effectiveness of vaccination resulted in over 88% reduction in symptomatic cases over a period of at least 5 months [16]. In addition, these studies further found that the strict adherence to safety guidelines, as well as the use of personal protective equipment largely reduced the overall spread of the virus and gave a sense of protection to healthcare workers including those working in COVID-19 assigned areas [14].

Regarding the diversity of the SARS-CoV-2 variants and strains, Hodcroft *et al.* [18] have been thoroughly tracking the appearance of new variants and strains, and with respect to Mexico. In 2020, an unidentified strain, referred to as "Other," predominated, followed by the B.1.1.519 strain emerged, notably carrying mutated versions of Variants of Concern, including S:T478K and S:P681H, all of which were related to high levels of hospitalization [17,18]. Subsequently, the Gamma and Delta strains, particularly the 21J variant, became dominant for most of 2021. Nevertheless, by the late 2021 and all of 2022, Omicron variants, particularly BA.1 and BA.5 emerged as the most prominent and successful strains, which while continuing to be highly transmissible did not seem to be of greater severity than previous variants [19]. Supplemental Figure 3 shows a graphic depiction of the major COVID-19 variants found in Mexico. From the perspective of children, data showed that fatality in young children was considerable, accounting for 5.19% of deaths amongst COVID-19-infected young children.

It is important to note that before any viable vaccine was available in Mexico, including those for other populations (particularly parents) the highest calculated fatality in COVID-19-positive young children was reached in 2020 with an estimated 10.37% of deaths. These results were similar when we compared them to the general population of Mexico. By July 31, 2020, total COVID-1-positive cases in Mexico had reached 424,467 with demises totaling 46,688 or approximately 10% [20]; this was considered the peak of the initial wave [5]. This early phase of the pandemic was a critical period as there was limited initial data on the behavior of COVID-19, as the WHO had only in March declared it an international epidemic as world cases had risen in a disproportionate manner [21]. As mentioned during this time, no available vaccines were yet available [4,22]. Worldwide efforts to mitigate the spread of the virus began with social distancing measures, as well as the use of personal protective equipment such as masks [4]. In Mexico, the government further implemented the suspension of non-essential activities and the Jornada Nacional de "Sana Distancia" or the social distancing initiative [23]. In addition, and with direct affectation to children, school closures were implemented, and activities were moved to online formats [24].

Fortunately, in Mexico, the first availability of vaccines was for older adults, as well as for the pregnant population, which began around 2021. A few months later, vaccines for adults 18 and older were widely available and by June 2021, vaccines for teens became accessible. All these efforts combined with previous measures helped in the efforts for disease management [25]. As a result, in 2021 the total fatality ratio for young children descended to 7.04%, and by the end of 2022 case fatality ratio was down to 2.11%. Although child vaccination was not approved until late 2022 and only for children 5 and older [26], the widespread vaccination campaigns targeting adult populations are likely responsible as positive contributors to the initial establishment of a potential herd immunity. As a higher proportion of adults in the population acquired immunity through vaccination or prior infection, leading to decreased opportunities for children to be exposed to the disease [27]. Potential herd immunity, along with the rise of less severe forms of the SARS-CoV-2 virus such as Omicron [28] may have helped mitigate the severity of COVID-19 cases in children. While the strategy in Mexico was aimed initially at protecting the older population, as they can potentially present higher risk of infections and death due to other comorbidities, there is an important case for strategizing vaccine interventions targeted to children. Children (teens) of school age, represent an important part of the population in Mexico [29], which can easily be accessible for an epidemiological intervention such as vaccination, which is aimed at reducing the spread of the virus [29], [30], [31]. Lin *et al.* [16] recently demonstrated that interventions, with mRNA bivalent vaccines at children aged 12 and under resulted in an important spread reduction, particularly in children aged 0 to 4 years there was an effectiveness of over 65%.

One remarkable observation is that hospitalizations in the general population and more significantly for COVID-19-positive young children arose from 2021-2022, having no consequence in the overall decline effects on death, as they were cut almost by half. While numerous reasons can exist for the overall general population including hospitalization because of incidence and other comorbidities without the threat of death, it is quite interesting that in COVID-19, hospitalizations more than doubled going from 398 to 900 or an increase of 126%, yet deaths only represented 2.11%. Is the increase in total cases a result of relaxing COVID-19 restrictions? Potentially, yes. We should state that, in the first quarter of 2022, and because of the positive effects of vaccination, safe distancing, usage of masks, and other measures implemented to mitigate COVID-19, several restrictions were lifted, giving more freedom of movement to the general population [32], hence potentially increasing human interactions and therefore potentiating the possible increase spread of COVID-19.

Earlier mentioned, more than half of patients passed within a week, above 77% within 2 weeks of hospitalization and the vast majority of cases were within the newborn infant group. As we focus on young children, those under 9 years, it is important to reaffirm that many of them lack the development of the fully developed immune system, making them susceptible to infections. The immune system of children gradually develops during infancy, with initial protection provided by immunoglobulin G antibodies passed from the mother transplacentally and through lactation. As these antibodies decline, young children become more vulnerable to infections [33]. Importantly, newborn infants may have impaired neutrophil function via reduced response to inflammatory stimuli, chemotaxis, and adhesion to endothelial cells, making them prone to infections. Monocytes and macrophages in infants have been reported to have lower levels of toll-like receptor 4 affecting innate signaling pathways and, consequently, compromising phagocytosis of potential pathogens [34]. Compared with blood from other age groups, cord blood contains a reduced quantity of myeloid-type dendritic cells. These cells secrete interleukin-12p70 in a smaller proportion in response to innate immune system-activating stimuli. Therefore, execution of T helper 1 and clusters of differentiations 8 T-cell responses is reduced compared to adults, correlating with an increased susceptibility to viral infections [35]. Newborn Plasmacytoid dendritic cells interferon secretion is also impaired upon exposure to different viruses, and this leads to a decreased ability to mount effective immune responses against viruses [36].

Finally, we should remark that certain comorbidities had a central role in relation to death in both the general and the COVID-19-positive populations. Particularly diabetes, intubation, and pneumonia seem to have dire consequences for patients. In our study, we found diabetes to be a comorbidity associated with demises in both the general population and the COVID-19-positive population. Ineffectively controlled diabetes greatly increases the risk of various types of infections, especially those of the respiratory tract [37]. A retrospective study found that suboptimal glycemic control in COVID-19-positive patients was associated with increased hospitalization for various types of infections in patients with type I and type II diabetes [38]. In particular, the angiotensin-converting enzyme 2 receptor, the binding receptor for SARS-CoV-2, has been shown to be overexpressed in lung cells of diabetic patients. This facilitates viral internalization and proliferation in these patients. Historically, diabetes mellitus has been recognized for its effects on the immune system. The immune system dysfunction seen in patients with COVID-19 and diabetes is attributed to the fact that diabetes causes a chronic hyperinflammatory state that can lead to excessive recruitment of macrophages, monocytes, and T cells. Overproduction of proinflammatory cytokines can ultimately damage lung tissue and endothelium. Therefore, alveolar dysfunction is also associated with diabetes as it may cause increased vascular permeability and decreased gas exchange, exacerbating pulmonary complications and increasing the need for mechanical ventilation in diabetic patients [37].

One of the major challenges in managing severe respiratory syndromes is both the prompt mobilization of oxygen to the body, and the potential to develop other related infections. Intubation, principally pediatric intubation, requires highly skilled trained health care professionals in the ICU. As a note, intubation using an endotracheal tube can have direct deleterious effects on the airways, including mucosal injury, reduced mucociliary function, and evasion of upper respiratory tract defenses such as the cough defense mechanism. One drawback of the endotracheal tube is that it may provide a novel environment for bacteria to attach and grow, promoting airway colonization and pneumonia. Airway mucosa abrasions can also create binding sites for bacteria and stimulate mucus secretion, leading to mucus deposition and potential sites for bacterial proliferation, this is primarily important in pediatric patients as within this population, their immune system is not yet fully developed, preventing them from having a higher degree of protection [39]. Whether pneumonia led to intubation or intubation caused nosocomial pneumonia, these conditions can significantly complicate the prognosis of patients with COVID-19, regardless of the disease's development.

An important strength of our study is that studies in children on the behavior of COVID-19 disease which presents infection rates, mortality, and symptomatology related to specific comorbidities, continue to be infrequent [40]. Additionally, we made an effort to include the available public data which spans over a five-wave (3 year) period to include as much information as possible. Nevertheless, our study is limited in the specific information registered and provided from the public database. Understandably, hospitalized young children are a vulnerable population, wherein considering comorbidities as well as COVID-19 becomes a complex problem. Therefore, an important weakness we find is that there is no information regarding the state of vaccination on any patient, even though vaccination efforts were set in place by early 2021. In addition, any information about treatment during hospitalization (if applicable) was also not provided. To best complete the overall picture, further analysis is warranted considering pharmacological intake (when applicable), more detailed information on aspects of comorbidities, social and family aspects including social, economic, and educational aspects of parents or guardians, and willingness to receive vaccination for parents, guardians, and children.

## Conclusion

Our goal was to present an overview of the conditions found in hospitalized young children for the metropolitan area of the valley of Mexico, as a representative population for Mexico's health care system. This work helps to preview what comorbidities and in what age groups they are more present and how they correlate, but also our information helps in understanding the health care management of the country. Vulnerable groups such as young children, present particular health care needs. Our study puts into light, these exact needs as related to comorbidities; it also permits us to understand the extent of death through the pandemic in young children. By now understanding the extent of death in different phases of the pandemic, particularly as different measures were set into place, we can have a clearer picture of how in the beginning, the death ramped up to >10%, and yet by having active pro-vaccine outreach programs, with high effective involvement of the community, death toll can be reduced by a great deal. These cultural behaviors related to vaccine acceptance and community involvement particularly with children can give a good perspective for managing future pandemics. This permits health authorities to make more informed decisions based on current events in the country. Tackling these decisions with detailed information can also help assist other countries in Latin America.

## Author contributions

GRPR and JFI were responsible for the project conceptualization. HFV, ENGT, and DFBC were responsible for data curation, GRPR and KGH were responsible for data analysis. MGSS and JFI wrote the original draft. All authors had access to all the data in the study and had final responsibility for the decision to submit for publication. All authors have read and agreed to the published version of the manuscript.

## Declarations of competing interest

The authors have no competing interests to declare.

## Appendix Supplementary materials

Image, image 1

Image, image 2

Image, image 3

Image, application 4

Image, application 5
