# Supplementary figures and images for: A panoramic view of hospitalized young children in the metropolitan area of the valley of Mexico during COVID-19
Source: IJID Reg. 2023 Oct 12;9:72–9. doi: 10.1016/j.ijregi.2023.10.004 (PMC10624577; doi:10.1016/j.ijregi.2023.10.004)

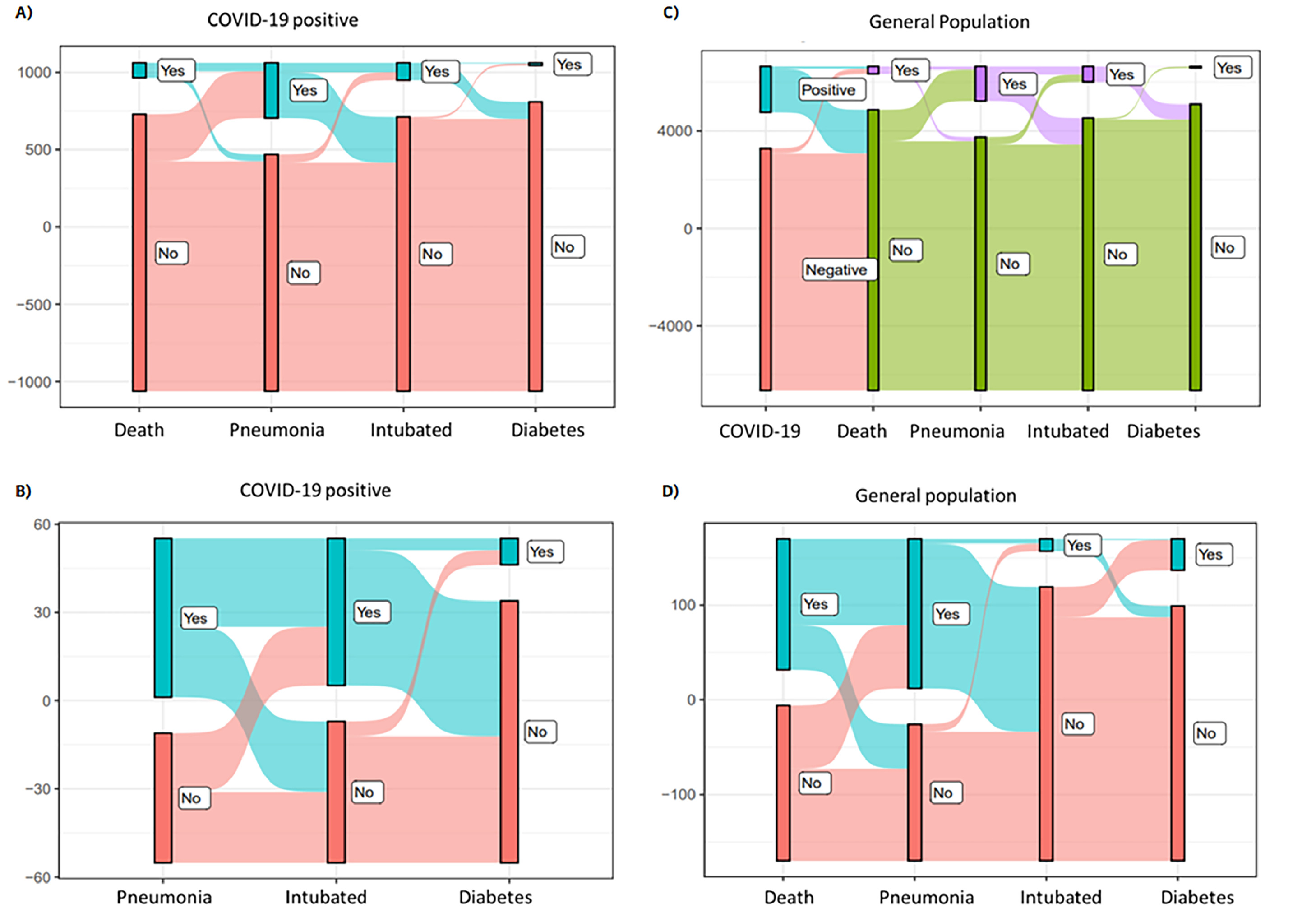

Supplement: Supplementary file 1 [file mmc1.jpg]

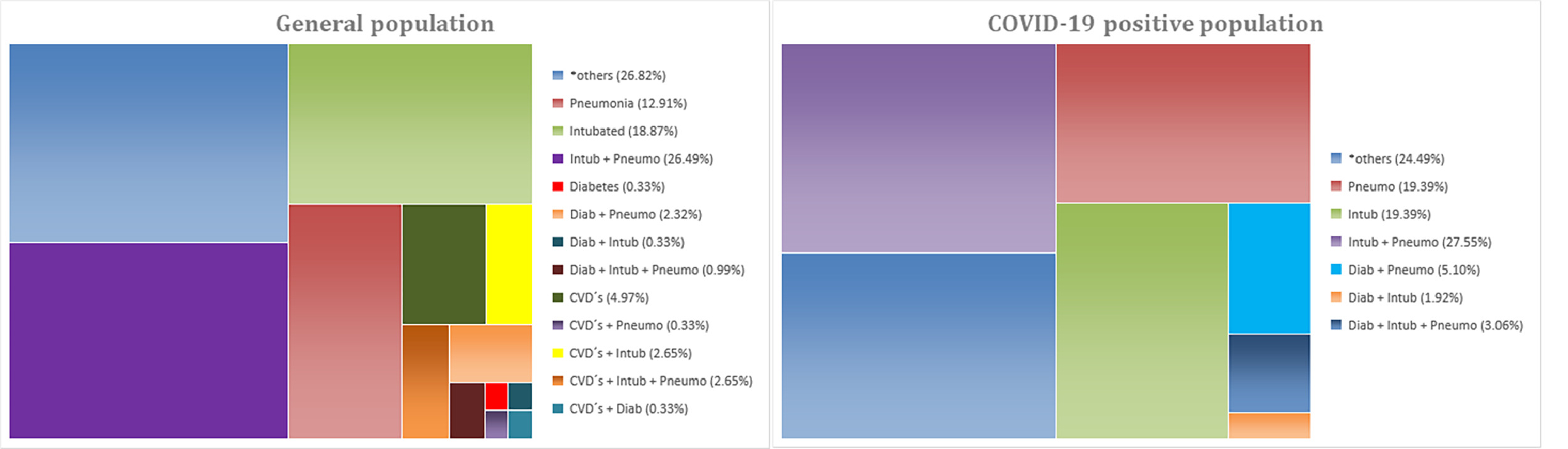

Supplement: Supplementary file 2 [file mmc2.jpg]

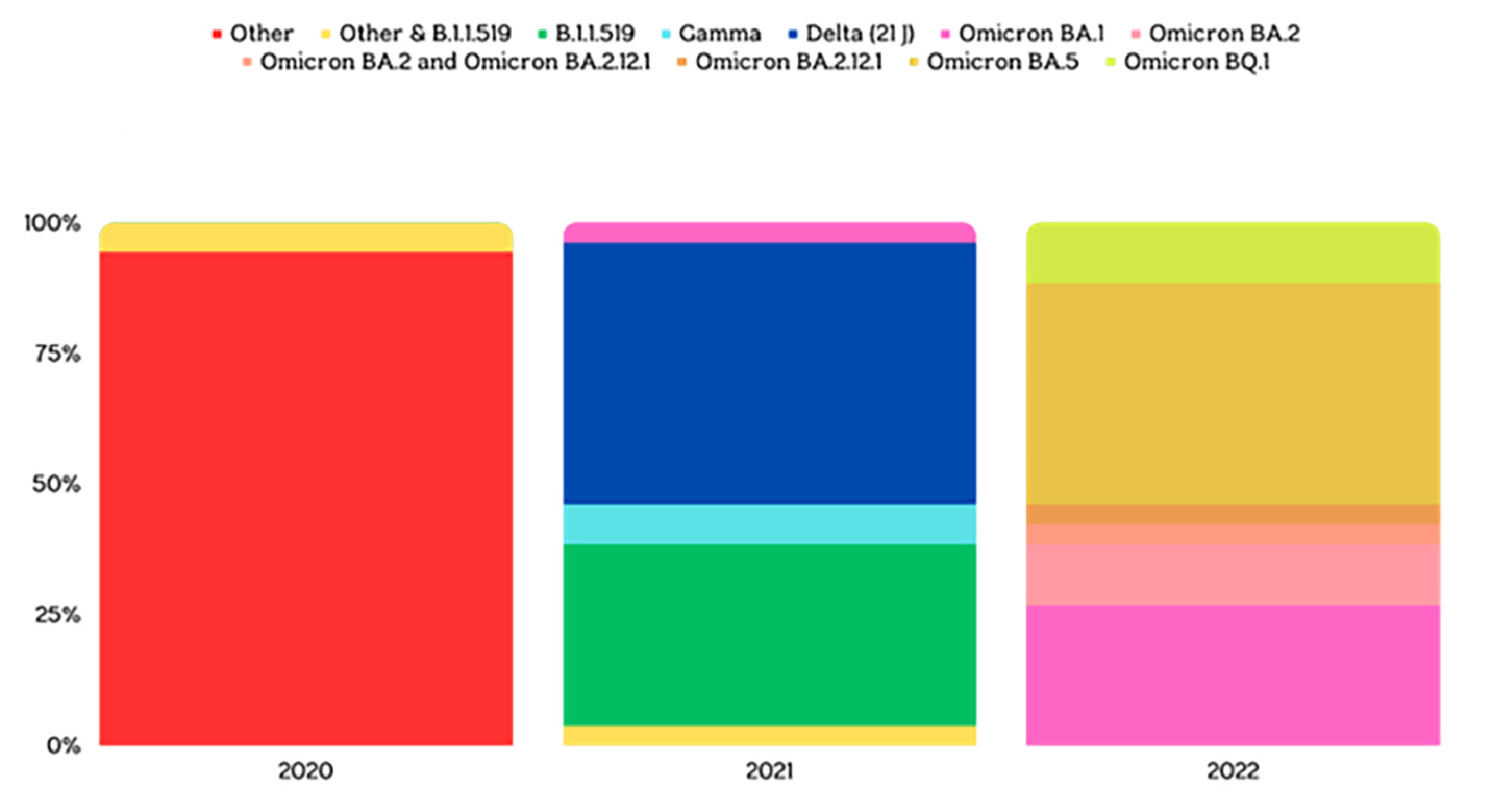

Supplement: Supplementary file 3 [file mmc3.jpg]
